# Dynamic linkages among economic development, environmental pollution and human health in Chinese

**DOI:** 10.1186/s12962-020-00228-6

**Published:** 2020-09-07

**Authors:** Ying Li, Tai-Yu Lin, Yung-Ho Chiu

**Affiliations:** 1 Business School, Sichuan University, Wangjiang Road No. 29, Chengdu, 610064 People’s Republic of China; 2 Department of Economics, Soochow University, No. 56, Kueiyang St., Sec. 1, Taipei, 100 Taiwan R.O.C.; 3 Department of Business Administration, National Cheng Kung University, No. 1, University Road, Tainan, 701 Taiwan R.O.C.

**Keywords:** Air pollutant, Data envelopment analysis, Economic efficiency, Energy consumption, Healthcare resource utilization efficiency

## Abstract

**Background:**

Research on the relationships between economic development, energy consumption, environmental pollution, and human health has tended to focus on the relationships between economic growth and air pollution, energy and air pollution, or the impact of air pollution on human health. However, there has been little past research focused on all the above associations.

**Methods:**

The few studies that have examined the interconnections between the economy, energy consumption, environmental pollution and health have tended to employ regression analyses, DEA (Data Envelopment Analysis), or DEA efficiency analyses; however, as these are static analysis tools, the analyses did not fully reveal the sustainable economic, energy, environmental or health developments over time, did not consider the regional differences, and most often ignored community health factors. To go some way to filling this gap, this paper developed a modified two stage Undesirable Meta Dynamic Network model to jointly analyze energy consumption, economic growth, air pollution and health treatment data in 31 Chinese high-income and upper-middle income cities from 2013–2016, for which the overall efficiency, production efficiency, healthcare resource utilization efficiency and technology gap ratio (TGR) for all input and output variables were calculated.

**Results:**

It was found that: (1) the annual average overall efficiency in China’s eastern region was the highest; (2) the production stage efficiencies were higher than the healthcare resource utilization stage efficiencies in most cities; (3) the high-income cities had lower TGRs than the upper–middle income cities; (4) the high-income cities had higher average energy consumption efficiencies than the upper-middle income cities; (5) the health expenditure efficiencies were the lowest of all inputs; (6) the high-income cities’ respiratory disease and mortality rate efficiencies were higher than in the upper–middle income cities, which had improving mortality rate efficiencies; and (7) there were significant regional differences in the annual average input and output indicator efficiencies.

**Conclusions:**

First, the high-income cities had higher average efficiencies than the upper-middle income cities. Of the ten eastern region high-income cities, Guangzhou and Shanghai had average efficiencies of 1, with the least efficient being Shijiazhuang. In the other regions, the upper-middle income cities required greater technology and health treatment investments. Second, Guangzhou, Lhasa, Nanning, and Shanghai had production efficiencies of 1, and Guangzhou, Lhasa, Nanning, Shanghai and Fuzhou had healthcare resource utilization efficiencies of 1. As the average production stage efficiencies in most cities were higher than the healthcare resource utilization stage efficiencies, greater efforts are needed to improve the healthcare resource utilization. Third, the technology gap ratios (TGRs) in the high-income cities were slightly higher than in the upper-middle income cities. Therefore, the upper-middle income cities need to learn from the high-income cities to improve their general health treatment TGRs. Fourth, while the high-income cities had higher energy consumption efficiencies than the upper-middle income cities, these were decreasing in most cities. There were few respiratory disease efficiency differences between the high-income and upper-middle income cities, the high-income cities had falling mortality rate efficiencies, and the upper-middle income cities had increasing mortality rate efficiencies. Overall, therefore, most cities needed to strengthen their health governance to balance economic growth and urban expansion. Fifth, the average AQI efficiencies in both the high-income and upper-middle income cities were higher than the average CO_2_ efficiencies. However, the high-income cities had lower average CO_2_ emissions and AQI efficiencies than the upper-middle income cities, with the AQI efficiency differences between the two city groups expanding. As most cities were focusing more on air pollution controls than carbon dioxide emissions, greater efforts were needed in coordinating the air pollution and carbon dioxide emissions treatments. Therefore, the following suggestions are given. (1) The government should reform the hospital and medical systems. (2) Local governments need to strengthen their air pollution and disease education. (3) High-income cities need to improve their healthcare governance to reduce the incidence of respiratory diseases and the associated mortality. (4) Healthcare governance efficiency needs to be prioritized in 17 upper-middle income cities, such as Hangzhou, Changchun, Harbin, Chengdu, Guiyang, Kunming and Xi’an, by establishing sound medical management systems and emergency environmental pollution treatments, and by increasing capital asset medical investments. (5) Upper-middle income cities need to adapt their treatment controls to local conditions and design medium to long-term development strategies. (6) Upper-middle income cities need to actively learn from the technological and governance experiences in the more efficient higher-income cities.

## Background

Global fossil fuel resources have been rapidly depleted since the beginning of the industrial revolution, and in the past few decades, climate change effects have become more apparent around the world. As a result, there has been an increased focus on energy efficiency and new clean energy technologies to reduce global carbon emissions. Because China is now the largest carbon dioxide emitter at around 28% of all global emissions, the Chinese government launched the “National Ambient Air Quality Standard”, which outlined aims to reduce its PM_2.5_ national emissions to 35 μg/m^3^ by 2030 [50]. The 13th Five-Year Plan, which was adopted on 15 March 2016, also included a goal to reduce sulfur dioxide and carbon dioxide emissions by 7% by 2040 [50]. Air pollutants and especially particulate matter have been found to contribute to increases in urban lung and cardiovascular diseases [32], with the World Health Organization (2018) reporting that in 2016, there were 4.2 million premature deaths due to PM_2.5_; 5 8% due to heart disease and stroke, 18% due to chronic obstructive pulmonary disease and acute lower respiratory infection, and 6% due to lung cancer. Because China has the world’s worst air quality, the Chinese government has begun to heavily invest in air quality improvements.

There has been significant research into the relationships between economic development, energy, and environmental pollution [17, 27, 48, 49, 52]), with some specifically focusing on energy and air pollution factors ([1, 8, 9, 12, 13, 18, 19, 26, 37, 48, 49, 54, 55]). The relationships between air pollution, health, and especially children’s health have also been major research foci [14, 16, 20, 21, 25, 31, 33, 36, 40, 46, 47, 51, 53, 56] and [6, 7, 15, 22, 23, 24, 30, 34, 38, 39]).

However, there has been little research on the interconnections between economic development, energy consumption, environmental pollution and health, or environmental pollution’s impact on social activities due to the health effects. The few studies that have examined these interconnections have tended to employ regression analyses, DEA (Data Envelopment Analysis), or static DEA efficiency tools that have lacked any dynamic considerations, which meant that the results did not fully reveal the sustainable economic, energy, environmental, and health developments over time. Therefore, past research has ignored the impact of social activities on human health, failed to reflect annual changes, and also failed to provide a comprehensive discussion on the connections between the economy, environmental pollution and health issues. Further, past research has only tended to examine the impact of environmental pollution such as CO_2_ and PM2.5 on first-stage economic production and have rarely considered the important AQI environmental indicators or discussed the relationships between production and health management.

Even though it is well known that different cities and different regions have different development levels, a majority of past studies have failed to provide such regional comparisons. While Feng et al. [14] recently employed a two-stage meta-frontier dynamic network data envelopment analysis (TMDN-DEA) model to explore energy consumption’s environmental pollution effects on child and adult mortality in 28 EU countries and 53 non-EU countries from 2010 to 2014, the research was mainly focused on the EU and non-EU countries. Therefore, to go some way to filling this gap, this study proposes a modified Undesirable Meta Dynamic Network model to explore the economic, energy, environmental, and human health efficiencies in 31 Chinese cities.

This research has two main contributions. First, this study explored the economic, energy and environmental pollution efficiencies across China and the associated government health expenditure and disease efficiencies. Second, to avoid any shortcomings associated with static analyses, carry-over impacts across the periods were included and the regional differences accounted for, for which a modified Undesirable Meta Dynamic Network model was developed to assess data from 2013–2016 in 31 Chinese cities in two stages; a production stage and a health treatment stage. In the production stage, labor and energy consumption were the inputs, GDP was the output, and CO_2_ (carbon dioxide) and the AQI (air quality index) emissions were the link variables between the production stage and health treatment stage, and in the second health treatment stage, health expenditure was the input, birth rate, respiratory disease rate, and mortality rate were the outputs, with the carryover being fixed assets.

The remainder of this paper is organized as follows. “Literature review” section gives the Literature review,  “Research method” section details the research method, “Empirical study” section examines the empirical results and provides an analytical discussion, and “Discussion” section gives the policy recommendations and managerial implications.

## Literature review

Research on the connections between economic development, energy, environmental pollution, and human health has tended to follow three main paths: the relationships between economic growth and air pollution; energy and air pollution efficiencies; and the impact of air pollution on human health.

### Relationships between economic growth and air pollution

Because the unfettered global focus on economic growth has led to a significant increase in environmental pollution, there has been a growing body of research focused on the relationships between economic growth and air pollution.

Georgiev and Mihaylo [17] tested the Environmental Kuznets Curve (EKC) hypothesis on four local and two global air pollutants and found that the EKC inverted U-shaped relationship between income and pollution did not hold for all gases, Xie et al. [52] found that an improvement in PM_2.5_ emissions would result in a growth in China’s GDP, Li et al. [27] conducted an environmental and economic analysis using a willingness to pay model, Wang et al. [48, 49] used panel smooth transition (PSTR) models to study the relationships between China’s economic growth and carbon dioxide emissions.

### Energy and air pollution efficiency

Energy consumption and economic development are inevitably linked; however, higher energy consumption generally results in a commensurate increase in air pollution; therefore, there has been a significant research focus on energy efficiencies. For example, Hu and Wang [19] analyzed the energy efficiencies in 29 Chinese administrative regions from 1995 to 2002 and found that Central China had the worst energy efficiency, Fang et al. [12] used DEA to study energy performances in China and the United States, and found that China’s technical efficiency was worse, and Choi et al. [8] explored low carbon dioxide efficiency in China using an SBM model. In other research, Liou and Hu [26] calculated the ecological total-factor energy efficiencies (ETFEE) in 30 Chinese regions from 2005 to 2009 using an SBM model, and found that there was a monotonic increasing relationship between regional ETFEE and China’s per capita GDP. In more recent studies, Zhang and Choi [55] employed an SBM model and found that most provinces had low energy efficiencies, Apergis et al. [1] found that the energy efficiencies in capital-intensive OECD countries were higher than in energy-intensive OECD countries, and Yao et al. [54] employed panel data and a meta-frontier non-radial Malmquist CO_2_ emissions performance index (MNMCPI) and found that the Chinese industrial sector had an average annual CO_2_ emissions growth rate of 5.53%, the average industrial sector carbon dioxide emissions in the eastern, central and western regions had declined, and the MCPI has overestimated the carbon dioxide emissions efficiencies.

More recently, Wang et al. [48, 49] examined China’s energy productivity from 1995 to 2012, and found that the capital and energy trend substitutions were similar to the labor and energy trend substitutions and that energy productivity changes were mainly affected by technological progress, Guo et al. [18] used a dynamic SBM DEA to study the energy efficiency in 26 OECD countries, finding that Canada and China had the best, Qin et al. [37] explored the energy efficiency of Chinese coastal areas and found that the energy efficiencies were lower when undesirable output was considered, and Feng et al. [13] explored the effect of industrial structural adjustment market-oriented reforms and the strengthening of environmental protection measures on China’s CO_2_ emissions efficiency. In other research, Emrouznejad and Yang [9] reviewed energy efficiency DEA research, Li and Lin [28] used bootstrap to survey total factor energy consumption in China, and Li et al. [29] found that high-income Chinese cities had higher technological efficiencies than low-income Chinese cities from 2013 to 2016.

### Impact of air pollution on human health

There has been substantial research into the effects of specific air pollutants because of the known adverse effects on community health. For example, Pope [36] found that a local steel mill was emitting 82% of all industrial $PM_{10}$(particulate matter) and that in some months from April 1985 to February 1988, the daily $PM_{10}$ levels exceeded 150 μg/m in the Utah Valley. Using a Poisson regression, Maheswaran et al. [31] examined the effects of NO_2_ (nitrogen dioxide) PM_10_ and CO (carbon monoxide) on stroke mortality and hospital admissions in Sheffield, United Kingdom from 1994 to 1998, and found that outdoor air pollution levels were associated with higher stroke mortality and hospital admission risks. Fischer et al. [16] found that long-term exposures to PM_10_ and NO_2_ were related to mortality in people over the age of 30 in the Netherlands, Lelieveld et al. [25] found that PM_2.5_ (particulate matter) caused 3.3% of annual worldwide premature deaths, and Wu et al. [51] concluded that exposure to particulate air pollution affected circulating antioxidant enzymes. In more recent studies, Yang et al. [53] found that all pollutants were positively correlated with prehypertension in northeast China in 2009, Vlaandern et al. [47] found that short-term exposure to air pollution interfered with blood metabolism, Shen et al. [40] found that the current AQI system was unable to accurately estimate the air pollution health risks, and Zhao et al. [56] found that of the 307 cyclists interviewed in Beijing in 2015 on heavily polluted days, most were low-income males over 30 years old or short-distance travelers. In related research, Ngo et al. [33] found that sandstorms were associated with per capita acute respiratory diseases, Torres et al. [46] found that sulfur dioxide and fine particles increased cardiovascular and respiratory diseases, Huang et al. [20] found that family income and education were negatively correlated with ambient air quality in Beijing in 2014, and Lua et al. [30] estimated that in 2017, 3800 out of 124,800 deaths in Hainan were due to PM_2.5_ exposure.

The impact of air pollution on children’s health has also been widely examined. For example, Feng et al. [14] employed a two-stage meta-frontier dynamic network DEA (TMDN-DEA) model to explore the influence of energy consumption and the associated environmental pollution on the mortality of children and adults in 28 EU countries and 53 non-EU countries from 2010 to 2014, Lee et al. [24] used a generalized additive model (GAM) time series analysis to explore the effect of multiple air pollutants (sulfur dioxide (SO_2_), nitrogen dioxide (NO_2_), ozone (O_3_), carbon monoxide (CO) and aerodynamic diameter (PM_10_)) on the health of the children under 15 years old in Seoul from 1997 to 1999, and found that nitrogen dioxide and ozone were the main contributors to childhood asthma. In more recent research, Chen et al. [6] found that PM_2.5_ and PM_10_ affected the health and lung function of primary school children, Fioravanti et al. [15] found that traffic air pollution had no effect on childhood obesity in children aged 4 and 8 years in Rome, and Knibbs et al. [22] found that exposure to outdoor NO_2_ affected the respiratory systems of children aged 7–11 in Australia. In other similar studies, Salavati et al. [39] found that air pollutants affected pregnant women’s health, Chen et al. [7] found that a higher air pollution exposure was associated with respiratory disease and impaired lung function prevalence in younger children in China, Nobles et al. [34] found that air pollution caused fetal growth restrictions, Landrigan et al. [23] found that air, water, soil and chemical pollution caused 940,000 child deaths worldwide, and Roberts et al. [38] found no associations between pollution exposure, air quality and mental health issues.

### Literature review summary

Most of the above research only focused on one or two aspects, with no studies having analyzed the relationships between economic development, energy, environmental pollution and human health. Therefore, to fill this gap, the method proposed in Feng et al. [14] was used to develop a modified Undesirable Meta Dynamic Network DEA model to examine the economic development, energy, environmental pollution and human health efficiencies in 31 Chinese cities in China, identify the regional differences, and provide suggestions for improvement.

## Research method

### Methodological framework

Past research on energy and environmental efficiencies ([19]) used labor, fixed assets and energy consumption as the inputs and GDP, CO_2_ and SO_2_ emissions as the outputs. This study used panel data from 31 of the most developed high-income and upper-middle income cities in China, the division for which was based on the World Bank’s classification for rich and poor countries, that is, the high-income economies had a GNI per capita of $12,056 or more, and the upper-middle income economies had a GNI per capita of between $3896 and $12,055.

From this calculation, the 31 sample cities were divided into 14 high-income cities: Beijing, Changsha, Fuzhou, Guangzhou, Hangzhou, Huhehot, Jinan, Nanchang, Nanjing, Shanghai, Shenyang, Tianjin, Wuhan, and Zhengzhou: and 17 upper-middle income cities: Chengdu, Changchun, Chongqing, Guiyang, Harbin, Haikou, Hefei, Kunming, Lanzhou, Lhasa, Nanning, Shijiazhuang, Taiyuan, Urumqi, Xian, Xining, Yinchuan. Data from 2013 to 2016 were extracted from the Statistical Yearbooks of China, the Demographics and Employment Statistical Yearbooks of China, and the Statistical yearbooks from each city. Air pollutant data were collected from China Environmental Protection Bureau reports.

In the first stage analysis of the energy and economic efficiencies in each city, labor and energy consumption were the input indicators and GDP was the output indicator, with the air quality index (AQI) and carbon dioxide (CO_2_) emissions being the link indicators, and in the second stage analysis of the government health expenditure efficiencies in each city, government health expenditure was the input indicator, and births, respiratory disease prevalence, and mortality rates were the output indicators, with the carryover being fixed assets.

The variables are explained in the following.

**Input variables**

**Labor**: Employees; the number of employees in each city at the end of each year; Unit = people.

**Fixed assets**: capital stock in each city calculated as the fixed assets investment in each city; Unit = 100 million CNY;

**Energy consumption**: total energy consumption in each city; Unit = 100 million tonnes.

**Output variables**

**GDP (Gross Domestic Product)**: The first stage of the research model in this study used gross domestic product (GDP) as the expected output, which is an important measure of the economic production in a country, a region or a city. Unit = 100 million CNY.

Link Production Stage and health stage variables:

**CO**_**2**_ **emissions**: the CO_2_ emissions in each city were estimated from the energy consumption breakdown by fuel category.

**Air quality Index (AQI)**: the measured pollutant concentrations for particulate matter: PM_2.5_, PM_10_, sulfur dioxide (SO_2_), Nitrogen Dioxide (NO_2_), Ozone(O_3_)and Carbon monoxide (CO), with the PM_2.5_ and PM_10_ being 24-h average concentrations.

**Second stage**: health treatment stage

**Input variables**: health expenditure

**Output variables**: Birth rate; respiratory diseases; mortality rate.

**Fig. 1:**
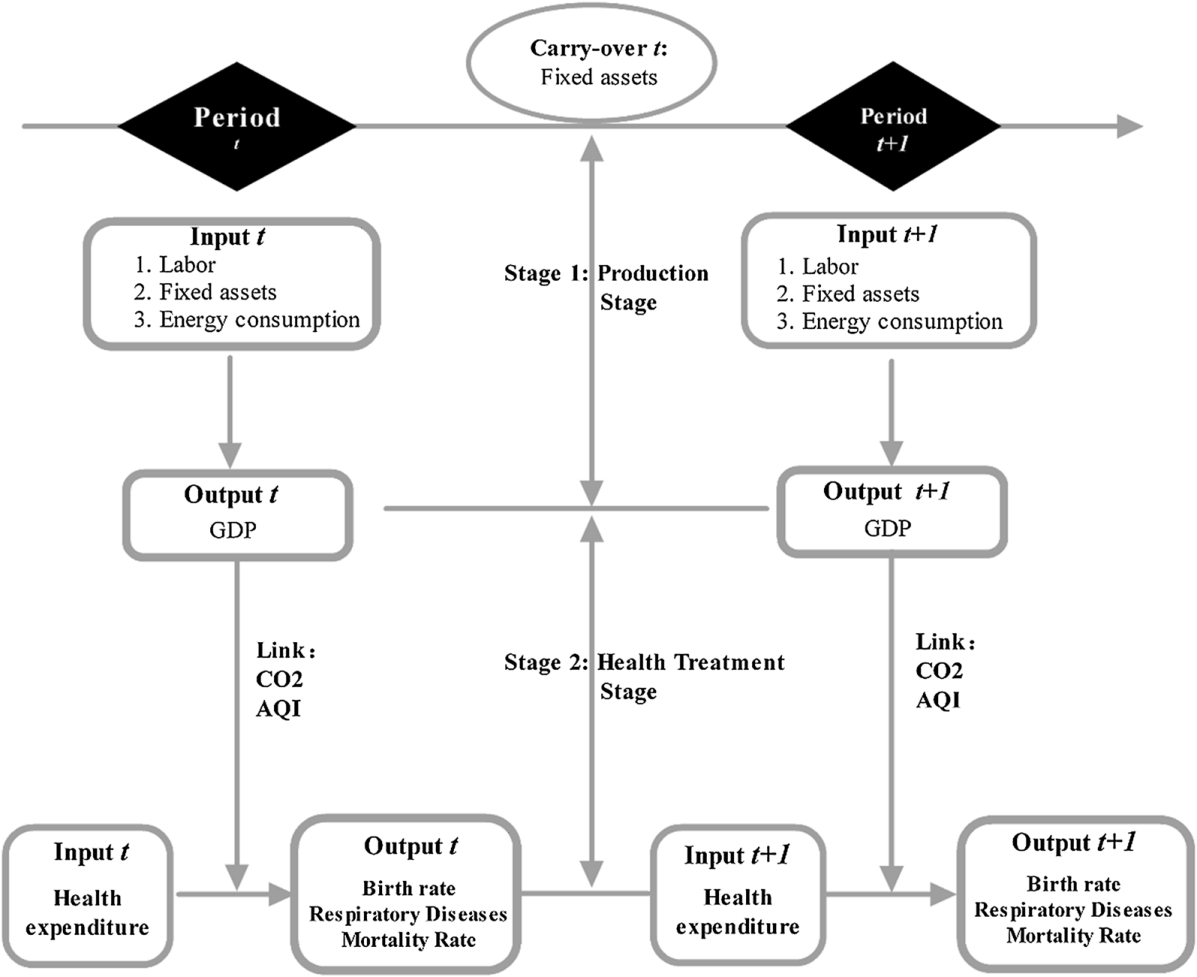
Network model

Figure 1 shows the Network Dynamic Model framework from Feng et al. [14] that was employed to explore the inter-period energy consumption, economic growth, air pollution and health efficiencies in 31 Chinese cities.

### Model parameterization

When developing the traditional DEA model, based on Farrell [10] ’s theory for a generalized mathematical linear programming model that could measure multiple inputs and outputs at constant returns to scale, Charnes et al. [5] developed the CCR model, which was extended in 1984 by Banker et al. [2] to the BCC variable returns to scale (VRS) model. However, both the CCR and BCC models measured radial efficiency, which assumed that the input items and/or output items proportionally increased or decreased. However, as this assumption is not applicable in all situations, Tone [41] proposed a Slacks-Based Measure (SBM) model in 2001 that used the slack variables as the basis for measurement, considered the slacks between the input and output items, and used a non-radial estimation method to find the single value (scalar) improvement space. Then, Färe et al. [11] proposed Network Data Envelopment Analysis to determine the optimal solution under the CCR and BCC models, which found that the production process was composed of many sub-production technologies. Tone and Tsutsui [42] then proposed a weighted slacks-based measure in which the links between the departments of the various decision-making units were used as the analysis basis for the network DEA model, with each department being regarded as a sub-DMU, after which the SBM model was used to find the most suitable solution. Unlike traditional DEA models, as these sub-production technologies were seen as “black boxes”, the Network DEA was applied to explore the impacts of the input allocations and intermediate goods on the production process. In a later network DEA model development, Tone and Tsutsui [43] adopted a dynamic method to evaluate the DMU efficiencies at different times, and then introduced a carryover to connect the various DMU stages in the different periods.

As the Dynamic DEA model measures operational efficiencies across multiple periods and the Network DEA analyzes the efficiency of individual departments, the limitations of traditional DEA models were overcome. Therefore, in 2014, Tone and Tsutsui proposed a weighted slacks-based Dynamic Network DEA, which treated each department as a sub-DMU, and acknowledged the links between the various decision-making unit departments and the carry-over activities between the different periods.

As this study considered both undesirable outputs and regional differences, Tone and Tsutsui’s [44] dynamic network SBM (Slacks-Based Measure) model was modified to an Undesirable Meta-Frontier Dynamic Network Model. Battese and Rao [3] and Battese et al. [4] proposed a meta-frontier model that was able to compare the technical efficiencies of different groups, after which O’Donnell et al. [35] established a meta-frontier model that accurately calculated meta-frontier and group efficiencies. However, most DEAs assume that all DMUs have the same technology levels, but as the DMUs in this study were in different geographical locations and were subject to varying regional policies, they had differing technology levels. Therefore, in reference to Feng et al.’s [14] framework and based on Tone and Tsutsui’s [44] Network Dynamic SBM, Tone and Tsutsui [45] and O’Donnell et al.’s [35] meta-frontier model, and by including the undesirable outputs, this paper developed an Undesirable Meta-Frontier Dynamic Network Model on Chinese data to establish economic, environmental and health models.

**Modified Undesirable Meta Dynamic Network model**

**Meta-frontier (MF)**

It is assumed that all units (N) are composed of DMUs in g groups (N = N_1_ + N_2_ +…. + N_G_), where y_rj_ and x_ij_ indicate the output item r (r = 1, 2, …, s) for item j (j = 1, 2, …, N) and input item i (i = 1, 2, …, m) for item j (j = 1, 2, …, N) under the meta-frontier. The DMU efficiency meta-frontier k is solved using the following linear programming (LP):

Suppose there are $n {\text{DMUs }}\left( {j = 1, \ldots ,{\text{n}}} \right)$, with each having $k$ divisions $\left( {k = 1, \ldots ,K} \right)$, and $T$ time periods $\left( {{\text{t}} = 1, \ldots ,{\text{T}}} \right)$. Each DMU has an input and output at time period $t$ and a carryover (link) to the next $t + 1$ time period.

**Inputs and outputs**

$X_{ijk}^{t} \in R_{ + } \left( {i = 1, \ldots ,m_{k} ; j = 1, \ldots ,n; k = 1, \ldots ,K; t = 1, \ldots ,T} \right)$ refers to input $i$ at time period $t$ for $DMU_{j}$ division $k$; $X_{ijk}^{t}$. In the first stage, the number of employees, fixed assets and energy consumption are used as the input variables, and in the second health expenditure treatment stage, government health expenditure is used as the input variable.

$Y_{rjk}^{t} \in R_{ + } \left( {r = 1, \ldots ,r_{k} ; j = 1, \ldots ,n; k = 1, \ldots ,K; t = 1, \ldots ,T} \right)$ refers to output r in time period $t$ for $DMU_{j}$ division $k$; $Y_{rjk}^{t}$. In the first stage, the GDP is the output, and in the second stage, the birth rate is the desirable output and respiratory disease and mortality rates are the undesirable outputs.

**Links**

$Z_{{j\left( {kh} \right)t}}^{t} \in R_{ + } (j = 1, \ldots ,n; l = 1, \ldots , L_{hk} ; t = 1, \ldots ,T$ are the period $t$ links from $DMU_{j}$ division $k$ to division $h$, with $L_{hk}$ being the number of $k$ to $h$ links; $Z_{{j\left( {kh} \right)t}}^{t}$. CO_2_ and the AQI are the link indicators in both the first production stage and the second health treatment stage.

**Carryovers**

$Z_{jkl}^{{\left( {t,t + 1} \right)}} \in R_{ + } \left( {j = 1, \ldots ,n; l = 1, \ldots , L_{k} ; k = 1, \ldots K; t = 1, \ldots ,T - 1} \right)$ are the carry-overs from $t$ to the $t + 1$ from $DMU_{j}$ division $k$ to division $h$, with $L_{k}$ being the number of carry-over items in division $k$; $Z_{jkl}^{{\left( {t,t + 1} \right)}}$. Fixed assets investment is the carry-over indicator in the production stage.

${\text{Linkin}}_{k}$ is the number of input links for each division k, ${\text{Linkout}}_{k}$ is the number of output links for each division k, ${\text{ngood}}_{k}$ indicates the number of desirable carry-overs for each division $k$, and ${\text{nbad}}_{k}$ indicates the number of undesirable carry-overs for each division $k$.

The meta-frontier $k$ for the DMU efficiency is solved using the following linear programming (LP):

**Objective function**

Overall efficiency:

1 $$\theta_{0}^{*} = \hbox{min} \frac{{\sum\nolimits_{{{\text{t}} = 1}}^{T} {W^{t} } \left[ {\sum\nolimits_{k = 1}^{K} {W^{k} \left[ {1 - \frac{1}{{m_{k} + linkin_{k} + ninput_{k} }}\left( {\sum\nolimits_{g = 1}^{G} {\sum\nolimits_{i = 1}^{{m_{k} }} {\frac{{S_{iok}^{t - } }}{{x_{iokg}^{t} }}} } + \sum\nolimits_{g = 1}^{G} {\sum\nolimits_{{(kl)_{l} = 1}}^{{linkin_{l} }} {\frac{{s_{{o(kh)_{l} in}}^{t} }}{{z_{{o(kh)_{l} ing}}^{t} }}} } + \sum\nolimits_{g = 1}^{G} {\sum\nolimits_{{k_{l} }}^{{ngood_{k} }} {\frac{{s_{{ok_{l} input}}^{(t,t + 1)} }}{{z_{{ok_{l} input}}^{(t,t + 1)} }}} } } \right)} \right]} } \right]}}{{\sum\limits_{t = 1}^{T} {W^{t} } \left[ {\sum\nolimits_{k = 1}^{K} {W^{k} \left[ {1 + \frac{1}{{r_{1k} + r_{2k} }}\left( {\sum\nolimits_{g = 1}^{G} {\sum\nolimits_{r = 1}^{{r_{1k} }} {\frac{{s_{rokgood}^{t + } }}{{y_{rokggood}^{t} }} + } } \sum\nolimits_{g = 1}^{G} {\sum\nolimits_{r = 1}^{{r_{2k} }} {\frac{{s_{rokbad}^{t - } }}{{y_{rokgbad}^{t} }}} } } \right)} \right]} } \right]}}$$

Subject to:

***Production stage***

$$x_{o1}^{t} = X_{1}^{t} \lambda_{1}^{t} + s_{1o}^{t - } \left( {\forall t} \right)$$;

$$y_{o1good}^{t} = Y_{1good}^{t} \lambda_{1}^{t} - s_{1ogood}^{t + } , \left( {\forall t} \right)$$;

$$\lambda_{1}^{t} \ge 0, s_{1o}^{t - } \ge 0, s_{1ogood}^{t + } \ge 0, \left( {\forall t} \right)$$;

$$Z_{{o\left( {12} \right)in}}^{t} = Z_{{\left( {12} \right)in}}^{t} \lambda_{k}^{t} + S_{{o\left( {12} \right)in}}^{t} \left( {\left( {12} \right)in} \right)$$

***Health treatment stage***

$$x_{o2}^{t} = X_{2}^{t} \lambda_{2}^{t} + s_{2o}^{t - } \left( {\forall t} \right)$$;

$$y_{o2good}^{t} = Y_{2good}^{t} \lambda_{2}^{t} - s_{2ogood}^{t + } \left( {\forall t} \right)$$;

$$y_{o2bad}^{t} = Y_{2bad}^{t} \lambda_{2}^{t} + s_{2obad}^{t - } \left( {\forall t} \right)$$;

$$\lambda_{2}^{t} \ge 0, s_{2o}^{t - } \ge 0, s_{2ogood}^{t + } \ge 0, s_{2obad}^{t - } \ge 0 \left( {\forall t} \right)$$;

$$e\lambda_{k}^{t} = 1\left( {\forall k, \forall t} \right)$$;

2 $$\begin{aligned} \sum\nolimits_{j = 1}^{n} {Z_{{jk_{1} \alpha }}^{{(t,\left( {t + 1} \right)}} \lambda_{jk}^{t} } & & = & & \sum\nolimits_{j = 1}^{n} {Z_{{jk_{1} \alpha }}^{{(t,\left( {t + 1} \right)}} \lambda_{jk}^{t + 1} \left( {\forall k, \forall t} \right)} \\ \sum\nolimits_{j = 1}^{n} {Z_{{jk_{1} \alpha }}^{{(t,\left( {t + 1} \right)}} \lambda_{jk}^{t} } & & & = & \mathop {\sum\nolimits_{j = 1}^{n} {Z_{{jk_{1} \alpha }}^{{(t,\left( {t + 1} \right)}} \lambda_{jk}^{t + 1} + S_{{ok_{l} input}}^{{\left( {t,t + 1} \right)}} (k_{l} = 1, \ldots ,ngood_{k} ;\;\forall k, \forall t);} }\limits_{{}}^{{}} \\ S_{{ok_{l} good}}^{{\left( {t,t + 1} \right)}} & & \ge 0,(\forall k, \forall t) \\ \end{aligned}$$

**(b) Period and division efficiencies**

The period and division efficiencies are as follows:

**(b1) Period efficiency**

3 $$\partial_{0}^{*} = \hbox{min} \frac{{\sum\nolimits_{k = 1}^{K} {W^{k} \left[ {1 - \frac{1}{{m_{k} + linkin_{k} + ninput_{k} }}\left( {\sum\nolimits_{g = 1}^{G} {\sum\nolimits_{i = 1}^{{m_{k} }} {\frac{{S_{iok}^{t - } }}{{x_{iokg}^{t} }} + } } \sum\nolimits_{g = 1}^{G} {\sum\nolimits_{{(kl)_{l} = 1}}^{{linkin_{l} }} {\frac{{s_{{o(kh)_{l} in}}^{t} }}{{z_{{o(kh)_{l} ing}}^{t} }}} } + \sum\nolimits_{g = 1}^{G} {\sum\nolimits_{{k_{l} }}^{{ninput_{k} }} {\frac{{s_{{ok_{l} input}}^{(t,t + 1)} }}{{z_{{ok_{l} ginput}}^{(t,t + 1)} }}} } } \right)} \right]} }}{{\sum\nolimits_{k = 1}^{K} {W^{k} \left[ {1 + \frac{1}{{r_{1k} + r_{2k} }}\left( {\sum\nolimits_{g = 1}^{G} {\sum\nolimits_{r = 1}^{{r_{1k} }} {\frac{{s_{rokgood}^{t + } }}{{y_{rokggood}^{t} }} + } } \sum\nolimits_{g = 1}^{G} {\sum\nolimits_{r = 1}^{{r_{1k} }} {\frac{{s_{rokbad}^{t - } }}{{y_{rokgbad}^{t} }}} } } \right)} \right]} }}$$

**(b2) Division efficiency**

4 $$\varphi_{0}^{*} = \hbox{min} \frac{{\sum\limits_{{{\text{t}} = 1}}^{T} {W^{t} } \left[ {1 - \frac{1}{{m_{k} + linkin_{k} + ninput_{k} }}\left( {\sum\nolimits_{g = 1}^{G} {\sum\nolimits_{i = 1}^{{m_{k} }} {\frac{{S_{iok}^{t - } }}{{x_{iokg}^{t} }} + } } \sum\nolimits_{g = 1}^{G} {\sum\nolimits_{{(kl)_{l} = 1}}^{{linkin_{l} }} {\frac{{s_{{o(kh)_{l} in}}^{t} }}{{z_{{o(kh)_{l} ing}}^{t} }} + } } \sum\nolimits_{g = 1}^{G} {\sum\nolimits_{{k_{l} }}^{{ninput_{k} }} {\frac{{s_{{ok_{l} input}}^{(t,t + 1)} }}{{z_{{ok_{l} ginput}}^{(t,t + 1)} }}} } } \right)} \right]}}{{\sum\limits_{t = 1}^{T} {W^{t} } \left[ {1 + \frac{1}{{r_{1k} + r_{2k} }}\left( {\sum\nolimits_{g = 1}^{G} {\sum\nolimits_{r = 1}^{{r_{1k} }} {\frac{{s_{rokgood}^{t + } }}{{y_{rokggood}^{t} }} + \sum\nolimits_{{{\text{g}} = 1}}^{\text{G}} {\sum\nolimits_{r = 1}^{{r_{2k} }} {\frac{{s_{rokbad}^{t - } }}{{y_{rokgbad}^{t} }}} } } } } \right)} \right]}}$$

**(b3) Division period efficiency**

5 $$\rho_{0}^{*} = \hbox{min} \frac{{\left[ {1 - \frac{1}{{m_{k} + linkin_{k} + ninput_{k} }}\left( {\sum\nolimits_{g = 1}^{G} {\sum\nolimits_{i = 1}^{{m_{k} }} {\frac{{S_{iok}^{t - } }}{{x_{iokg}^{t} }} + } } \sum\nolimits_{g = 1}^{G} {\sum\nolimits_{{(kl)_{l} = 1}}^{{linkin_{l} }} {\frac{{s_{{o(kh)_{l} in}}^{t} }}{{z_{{o(kh)_{l} ing}}^{t} }} + \sum\nolimits_{g = 1}^{G} {\sum\nolimits_{{k_{l} }}^{{ninput_{k} }} {\frac{{s_{{ok_{l} input}}^{(t,t + 1)} }}{{z_{{ok_{l} ginput}}^{(t,t + 1)} }}} } } } } \right)} \right]}}{{\left[ {1 + \frac{1}{{r_{1k} + r_{2k} }}\left( {\sum\nolimits_{g = 1}^{G} {\sum\nolimits_{r = 1}^{{r_{1k} }} {\frac{{s_{rokgood}^{t + } }}{{y_{rokggood}^{t} }} + \sum\nolimits_{{{\text{g}} = 1}}^{\text{G}} {\sum\nolimits_{r = 1}^{{r_{2k} }} {\frac{{s_{rokbad}^{t - } }}{{y_{rokgbad}^{t} }}} } } } } \right)} \right]}}$$

From the above, using the meta-frontier model, the overall efficiency, period efficiency, division efficiency, and division period efficiency can be obtained.

**Group-frontier (GF)**

As each DMU under the group frontier chooses the most favorable final weighted output, the DMU efficiencies under the group frontier are solved using the following equations.

**(a) Objective function**

Overall efficiency

6 $$\theta_{0}^{*} = \hbox{min} \frac{{\sum\nolimits_{t = 1}^{T} {W^{t} \left[ {\sum\nolimits_{k = 1}^{K} {W^{k} \left[ {1 - \frac{1}{{m_{k} + linkin_{k} + ninput_{k} }}\left( {\sum\nolimits_{i = 1}^{{m_{k} }} {\frac{{S_{iok}^{t - } }}{{x_{iok}^{t} }}} + \sum\nolimits_{{(kh)_{l} = 1}}^{{linkin_{k} }} {\frac{{s_{{o(kh)_{l} in}}^{t} }}{{z_{{o(kh)_{l} in}}^{t} }} + \sum\limits_{{k_{l} }}^{{ninput_{k} }} {\frac{{s_{{ok_{l} input}}^{(t,t + 1)} }}{{z_{{ok_{l} input}}^{(t,t + 1)} }}} } } \right)} \right]} } \right]} }}{{\sum\nolimits_{t = 1}^{T} {W^{t} \left[ {\sum\nolimits_{k = 1}^{K} {W^{k} \left[ {1 + \frac{1}{{r_{1k} + r_{2k} }}\left( {\sum\nolimits_{r = 1}^{{r_{1k} }} {\frac{{s_{rokgood}^{t + } }}{{y_{rokgood}^{t} }} + \, \sum\nolimits_{{{\text{r}} = 1}}^{{r_{2k} }} {\frac{{s_{rokbad}^{t - } }}{{y_{rokbad}^{t} }}} } } \right)} \right]} } \right]} }}$$

**(b) Period and division efficiencies**

The period and division efficiencies are as follows

**(b1) Period efficiency**

7 $$\partial_{0}^{*} = \hbox{min} \frac{{\sum\nolimits_{k = 1}^{K} {W^{k} \left[ {1 - \frac{1}{{m_{k} + linkin_{k} + ninput}}\left( {\sum\nolimits_{i = 1}^{{m_{k} }} {\frac{{S_{iok}^{t - } }}{{x_{iok}^{t} }}} + \sum\nolimits_{{(kh)_{l} = 1}}^{{linkin_{k} }} {\frac{{s_{{o(kh)_{l} in}}^{t} }}{{z_{{o(kh)_{l} in}}^{t} }} + \sum\limits_{{k_{l} }}^{{ninput_{k} }} {\frac{{s_{{ok_{l} input}}^{(t,t + 1)} }}{{z_{{ok_{l} input}}^{(t,t + 1)} }}} } } \right)} \right]} }}{{\sum\nolimits_{k = 1}^{K} {W^{k} \left[ {1 + \frac{1}{{r_{1k} + r_{2kk} }}\left( {\sum\nolimits_{r = 1}^{{r_{1k} }} {\frac{{s_{rokgood}^{t + } }}{{y_{rokgood}^{t} }} + \, \sum\nolimits_{{{\text{r}} = 1}}^{{r_{2k} }} {\frac{{s_{rokbad}^{t - } }}{{y_{rokbad}^{t} }}} } } \right)} \right]} }}$$

**(b2) Division efficiency**

8 $$\varphi_{0}^{*} = \hbox{min} \frac{{\sum\nolimits_{t = 1}^{T} {W^{t} \left[ {1 - \frac{1}{{m_{k} + linkin_{k} + ninput_{k} }}\left( {\sum\nolimits_{i = 1}^{{m_{k} }} {\frac{{S_{iok}^{t - } }}{{x_{iok}^{t} }}} + \sum\nolimits_{{(kh)_{l} = 1}}^{{linkin_{k} }} {\frac{{s_{{o(kh)_{l} in}}^{t} }}{{z_{{o(kh)_{l} in}}^{t} }} + \sum\nolimits_{{k_{l} }}^{{ninput_{k} }} {\frac{{s_{{ok_{l} input}}^{(t,t + 1)} }}{{z_{{ok_{l} input}}^{(t,t + 1)} }}} } } \right)} \right]} }}{{\sum\nolimits_{t = 1}^{T} {W^{t} \, \left[ {1 + \frac{1}{{r_{1k} + r_{2k} }}\left( {\sum\nolimits_{r = 1}^{{r_{1k} }} {\frac{{s_{rokgood}^{t + } }}{{y_{rokgood}^{t} }} + \, \sum\nolimits_{{{\text{r}} = 1}}^{{r_{2k} }} {\frac{{s_{rokbad}^{t - } }}{{y_{rokbad}^{t} }}} } } \right)} \right]} }}$$

**(b3) Division period efficiency**

9 $$\rho_{0}^{*} = \hbox{min} \frac{{1 - \frac{1}{{m_{k} + linkin_{k} + ninput_{k} }}\left( {\sum\nolimits_{i = 1}^{{m_{k} }} {\frac{{S_{iok}^{t - } }}{{x_{iok}^{t} }}} + \sum\nolimits_{{(kh)_{l} = 1}}^{{linkin_{k} }} {\frac{{s_{{o(kh)_{l} in}}^{t} }}{{z_{{o(kh)_{l} in}}^{t} }} + \sum\nolimits_{{k_{l} }}^{{ninput_{k} }} {\frac{{s_{{ok_{l} input}}^{(t,t + 1)} }}{{z_{{ok_{l} input}}^{(t,t + 1)} }}} } } \right)}}{{\left( {1 + \frac{1}{{r_{1k} + r_{2k} }}\left( {\sum\nolimits_{r = 1}^{{r_{1k} }} {\frac{{s_{rokgood}^{t + } }}{{y_{rokgood}^{t} }} + \, \sum\nolimits_{r = 1}^{{r_{2k} }} {\frac{{s_{rokbad}^{t - } }}{{y_{rokbad}^{t} }}} } } \right)} \right)}}$$

From the above results, the overall efficiency, the period efficiency, the division efficiency and division period efficiency are obtained.

**Technology gap ratio (TGR)**

As the meta-frontier model contains g groups, the technical efficiency of the meta-frontier (MFE) is less than the technical efficiency of the group frontier (GFE); therefore, the ratio value or technology gap ratio (TGR) is:

10 $${\text{TGR}} = \frac{{\rho^{ *} }}{{\rho_{o}^{ *g} }} = \frac{MFE}{GFE}$$

### Energy consumption, Health expenditure, Respiratory Diseases, Mortality Rate, CO_2_ and AQI Efficiencies

Hu and Wang’s [19] total-factor energy efficiency index was used to overcome any possible biases in the traditional energy efficiency indicators, for which there were eight key efficiency models; energy, environment, health expenditure, respiratory diseases, mortality rate, CO_2_ emissions, and the AQI. In this study, “I” represents area and “t” represents time.

The efficiency models are defined in the following:

11 $${\text{Inputefficiency}} = \frac{\text{Target input}}{\text{Actual input}}$$

12 $${\text{Undesirable output efficiency }} = \frac{\text{Target Undesirable output}}{\text{Actual Undesirable output}}$$

If the target inputs equal the actual inputs, then the efficiencies are 1, which indicates overall efficiency; however, if the target inputs are less than the actual inputs, then the efficiencies are less than 1, which indicates overall inefficiency.

If the target undesirable outputs are equal to the actual undesirable outputs, then the efficiencies are 1, indicating overall efficiency; however, if the target undesirable outputs are less than the actual undesirable outputs, then the efficiencies are less than 1, indicating overall inefficiency.

## Empirical study

### Statistical analysis of the inputs and outputs

Table 1 shows the average input–output indicator quantities in each Chinese region, from which it can be seen that there are large regional differences.

**Table 1:** Input and output variables from 2013–2016 by region

Cities by region	Labor				Fixed assets			
	2013	2014	2015	2016	2013	2014	2015	2016
East	11699010.00	11844340.00	11963880.00	12088240.00	4830.72	5391.81	5898.81	6331.43
Central	7284183.33	7377433.33	7503350.00	7620033.33	4047.34	4710.62	5425.93	5642.37
Northeast	8578700.00	8568300.00	8952350.00	8956250.00	5004.07	4888.20	4735.23	3794.10
West	7158463.64	7207287.82	7871963.64	8050936.36	3062.44	3504.51	3823.97	4271.25

Cities by regions	Energy consumption				GDP			
	2013	2014	2015	2016	2013	2014	2015	2016
East	5486.35	5543.14	5533.04	5403.25	10363.64	11230.38	12071.21	13126.46
Central	2710.46	2842.72	2862.06	1797.11	5475.23	6008.49	6521.09	7135.90
Northeast	2692.40	1831.23	1704.54	1725.09	5583.27	5927.07	6184.50	6441.80
West	2392.18	2326.52	2479.46	2451.69	3717.34	4108.43	4447.69	4861.24

Cities by regions	Carbon dioxide				AQI			
	2013	2014	2015	2016	2013	2014	2015	2016
East	14310.43	14071.31	13542.47	12285.41	156	95	86	79
Central	7742.42	9263.67	9482.40	6461.84	178	103	94	90
Northeast	6926.98	7000.23	6703.04	7237.43	168	89	99	75
West	7477.08	6849.72	6774.11	7240.09	126	85	78	79

The labor force indicators in Table 1 show that the urban labor average in the eastern region was the highest and was rising in all periods to 2016. The urban average inputs in the northeastern region were also rising with some fluctuations in all period to 2016. However, the labor inputs were relatively small in the eastern and western regions, with the average labor input in the western region being the lowest of the four regions in 2013 and 2014 but higher than the central region in the subsequent 2 years and showing a straight upward trend to the its highest level in 2016. Although the labor input in the central region also increased slightly, the input in 2015 and 2016 was the lowest of the four regions.

In 2013, the urban fixed assets average in the northeast was the highest of the four regions; however, after that time, the average fell from 2014 to 2016. The largest fixed assets increases were in the eastern region, which from 2014 to 2016 was the highest of all four regions. While the fixed assets input in the western region also continued to rise, the average urban fixed assets input from 2013 to 2015 was the smallest of the four regions, but in 2016 was higher than in the northeast. In general, the differences between the regions were expanding.

The eastern region had the largest energy consumption inputs in all time periods, the western region had the smallest in 2013, but from 2014 to 2016, the northeast region had the smallest and declining energy consumption inputs, with i s lowest level in all 4 years being in 2016.

The highest urban GDP average in all 4 years was in the eastern region, and even though the urban GDP average was rising in the western region, it was the lowest of the four regions in all 4 years.

Even though the carbon dioxide emissions in the eastern region were showing a linear downward trend in all 4 years, in 2016, they were still much higher than in the other regions. The carbon dioxide emissions were the lowest in the western region in 2014, but the lowest in the northeastern region in 2013, 2015 and 2016. The carbon dioxide emissions in the northeastern region were also rising slightly and the gap between the regions was narrowing.

The highest air pollution index value was in the central region in 2013, 2014 and 2016; however, in 2015, the average air pollution index in the northeastern region was higher than in the other regions. The lowest average urban air pollution index was in the western region from 2013 to 2015, but in 2016, it was the lowest in the northeastern region. The air pollution index in all four regions had a downward trend, indicating that the air quality in most regions was improving, with only the western region rebounding slightly in 2016.

### Results and analysis

#### Overall efficiency analysis

This study first compared the overall efficiencies in each city from 2013 to 2016, after which the first production stage efficiencies and the second healthcare resource utilization stage efficiencies were compared.

The overall efficiency was the output divided by the input, which was then expressed as a percentage; therefore, a perfect process would have an efficiency of 100%.

Appendix 1 shows the overall efficiency in each city from 2013 to 2016, from which it can be seen that Guangzhou, Lhasa, and Shanghai had overall efficiencies of 1 for all 4 years, Beijing had an overall efficiency of 1 in the first year, which dropped to below 0.6 in 2015 and 2016, Nanning had an overall efficiency of 1 for the first 3 years, which in the last year dropped to around 0.68, and Jinan’s efficiency was poor in the first 3 years, but reached 1 in the final year. Fuzhou, Haikou, and Urumqi had better overall efficiencies than the other cities; however, Urumqi’s efficiency was lower in 2015 at 0.6 but higher than 0.8 in the other years. These results were similar to the empirical results in Choi et al. [8] and Zhang and Choi [55]. In general, the overall efficiencies in most provinces needed improvement.

In most other cities, the overall efficiencies over the 4 years were below 0.6. Shijiazhuang had the lowest, with its highest efficiency in the 4 years being less than 0.3, the best annual efficiency in Taiyuan was less than 0.4 in 2013, Guiyang, Chengdu, Lanzhou, Kunming, Xian, and Xining’s had efficiencies of around 0.4, and Changchun, Harbin, Hangzhou, Hefei, Huhehot, Nanchang, Nanjing, Wuhan and Zhengzhou had efficiencies ranging from 0.4 to 0.6; therefore, most of these cities needed efficiency improvements.

Only six cities: Changsha, Chongqing, Hefei, Jinan, Shenyang, and Taiyuan: had increasing efficiencies, five of which were middle-income cities. Jinan’s efficiency, however, rose from around 0.4 to 1, and Hefei’s rose from below 0.5 in 2013 to close to 0.9 in 2016. Over the period, 23 cities saw decreasing efficiencies, with Beijing, Nanning and Wuhan experiencing the largest declines. Wuhan’s overall efficiency in 2013 was around 0.9, which dropped to only 0.4 in 2016, and Beijing’s overall efficiency in 2013 was 1, which by 2016 had fallen to around 0.5.

In reference to the empirical framework in Feng et al. [14], Table 2 shows the annual efficiencies in China’s eastern, central, northeastern and western regions. The annual average overall efficiency in the eastern region was the highest, but at 0.665, there was a great need for improvements. The average overall efficiency in the western region was significantly higher than in the central and northeastern regions in 2013 and 2015, but fluctuated and declined and by 2016 was lower than the central region for the first time. Of the four regions, the lowest average total efficiency was in the northeastern region at only 0.5, and had a fluctuating downward trend, with the lowest being in 2016 at only 0.381; therefore, significant improvements were needed. The annual average overall efficiency in the central region also had a fluctuating downward trend; however, there was a significant rise in 2016 to be second highest behind the eastern region, which was in line with the empirical results in Yao et al. [54]. Overall, all regions required overall efficiency improvements.

**Table 2:** Average overall efficiencies from 2013–2016 by region

Cities by regions	2013	2014	2015	2016
East	0.665	0.690	0.607	0.665
Central	0.535	0.486	0.465	0.502
Northeast	0.428	0.480	0.409	0.381
West	0.548	0.563	0.489	0.495

#### Production efficiency and healthcare resource utilization efficiency analyses

The significant regional economic growth and social development differences meant that there were large production and health treatment stage efficiencies differences between the cities (see Appendix 2). Guangzhou, Lhasa, Nanning, and Shanghai had production stage efficiencies of 1, and Guangzhou, Lhasa, Nanning, Shanghai and Fuzhou had heath treatment stage efficiencies of 1. Generally, the production stage efficiencies in most cities were higher than the **healthcare resource utilization** stage efficiencies. A detailed analysis of the two-stage efficiencies is given in the following.

i. Production efficiency

Production efficiency is when an economic system is unable to produce any more of one good without sacrificing the production of another good or without improving the production technology. In other words, production efficiency is when a good or a service is produced at the lowest possible cost. In simple terms, production efficiency is illustrated on a production possibility frontier on which all points on the curve are indicators of productive efficiency. However, equilibrium may be productively efficient without being allocative efficient, that is, it may result in a goods distribution in which the social welfare is not maximized.

A city/region’s production efficiency is when all services and enterprises within a city operate using best-practice technological and managerial processes and there are no further reallocations that can result in greater output with the same inputs and production technology. By improving these processes, however, an economy or a business can extend its production possibility frontier outward so that the efficient production yields a greater output than previously.

Twenty-one of the 31 cities had higher production stage efficiencies than healthcare resource utilization stage efficiencies, which indicated that these cities needed to place a greater focus on health treatment governance.

While Nanchang, Nanjing, Jinan and Beijing had production stage efficiencies over 0.8 for 2 years, 3 years and 4 years respectively, except for Lanzhou, Shijiazhuang, Taiyuan, and Xining, which had production stage efficiencies of less than 0.4, most other cities had production stage efficiencies between 0.4 and 0.8.

Hefei, Huhehot, Jinan, Nanjing, Taiyuan, and Xining had increasing production efficiencies, with the largest increase being in Jinan from below 0.6 in 2013 to 1 in 2016. The production stage efficiencies in the other 21 cities fell, with the biggest declines being in Fuzhou, Urumqi, and Zhengzhou to around 0.2. These results were similar to the empirical results in Wang et al. [48, 49], in which it was found that most regions had low production stage efficiencies and large regional differences.

ii. Healthcare resource utilization efficiency

The average healthcare resource utilization efficiencies were far lower than the average production stage efficiencies. Fuzhou, Haikou, and Urumqi had higher healthcare resource utilization efficiency stage efficiencies than production stage efficiencies in all 4 years, and Lanzhou, Yinchuan and Xining had higher healthcare resource utilization efficiencies than production stage efficiencies in 3 years. Therefore, the local governments in these ten cities should focus more resources on improving their production efficiencies.

Besides the five cities that had healthcare resource utilization efficiencies of 1 in all 4 years, the efficiencies in the other cities were generally low. Fifteen upper-middle income cities: Changchun, Chengdu, Guiyang, Harbin, Hangzhou, Huhehot, Kunming, Nanchang, Nanjing, Shenyang, Shijiazhuang, Taiyuan, Tianjin, Xian, and Zhengzhou: had healthcare resource utilization efficiencies below 0.4, with eight of these: Changchun, Chengdu, Harbin, Nanchang, Shijiazhuang, Tianjin, Xian and Zhengzhou: having healthcare resource utilization efficiencies below 0.2.

Table 3 shows the average annual production and health treatment stage efficiencies in the four regions. The highest annual average production stage efficiency was in the eastern region, which rose marginally over the 4 years. The central region had an average production stage efficiency just below the eastern region in 2013 and 2014, but it began falling in 2015, when it was slightly lower than in the northeast. The average efficiency in the central region had a fluctuating downward trend, with the lowest efficiency being in 2016. Of the four regions, the western region had the lowest average production stage efficiency, which had a downward fluctuating trend to its lowest in 2016 of only 0.569. Although the production stage efficiency in the northeast region was higher than in the western region and in 2015 was higher than both the western and central regions, in 2016 it fell to only 0.563, which was lowest of the four regions.

**Table 3:** Average production efficiency and healthcare resource utilization efficiency from 2013–2016 by region

Cities by regions	Production efficiency				Healthcare resource utilization efficiency			
	2013	2014	2015	2016	2013	2014	2015	2016
East	0.758	0.749	0.751	0.774	0.572	0.630	0.463	0.557
Central	0.724	0.731	0.721	0.630	0.346	0.244	0.208	0.374
Northeast	0.713	0.730	0.732	0.563	0.144	0.230	0.085	0.199
West	0.598	0.620	0.595	0.569	0.498	0.505	0.383	0.421

The regional health treatment stage annual average efficiencies varied widely across the four regions. The eastern region had the highest efficiency, followed by the western region, the central region and the northeastern region, which dropped to only 0.085 in 2015 and had a rebound in 2016 to about 0.2, its highest point in the 4 years. The health treatment stage annual average efficiency in the central region was lower than either the eastern or western region and had a U-shaped change, rising to its highest point in 4 years in 2016 at 0.374. However, in 2014 and 2015, the central region’s health treatment stage annual average efficiency was lower than 0.25, which was only slightly better than the northeast region; therefore, there were significant improvements needed.

#### Average overall efficiency and average TGR comparison between the city groups

Battese and Rao [3] stated that “the technology gap ratio indicates the technology gap for the given group according to currently available technology for firms in that group, relative to the technology available in the whole industry”.

To more clearly analyze the regional differences, the high-income and upper-middle-income city overall efficiencies and average TGRs were compared (Table 4).

**Table 4:** TGRs and efficiency from 2013–2016 for the high-income and upper-middle income cities

Cities by income	TGR				Efficiency			
	2013	2014	2015	2016	2013	2014	2015	2016
High-income	0.731	0.802	0.718	0.708	0.632	0.656	0.565	0.600
Upper-middle income	0.746	0.777	0.767	0.721	0.522	0.520	0.473	0.491
Wilcoxon test	0.354	0.271	0.211	0.437	0.040**	0.013**	0.024**	0.045**

**For the one-tailed test, the confidence interval 0.05 was significant

From Table 4, it can be seen that the high-income cities had higher average efficiencies than the middle-income cities, but there were some variances. The average efficiency in the high-income cities increased from 0.63 in 2013 to around 0.66 in 2014, declined in 2015 to around 0.57, and rebounded slightly in 2016 to 0.60. However, the upper-middle-income cities as a whole had lower average overall efficiencies. The average overall efficiency decreased slightly from 0.52 in 2013 to 0.51 in 2014, fell to 0.47 in 2015 and rose slightly to 0.49 in 2016; however, the overall efficiency decline was slightly less than in the high-income cities. The Wilcoxon Test indicated that the differences in the high-income and upper-middle income city efficiencies were significant at 5% from 2013 to 2016, which indicated that the efficiencies in the high-income cities were significantly better than in the upper-middle income cities.

The average TGRs were similar in the high-income and upper- middle income cities, with the average TGR in the high-income cities being only slightly higher at 0.8 in 2014 than the upper-middle income cities. However, in 2015 and 2016, the average TGR in the upper-middle income cities was slightly higher than in the high-income cities, indicating that the TGR was falling in the high-income cities and rising in the upper-middle income cities, and that the TGR gap between the two city types was narrowing. The Wilcoxon Test found that the TGR differences between the high-income and upper-middle income cities was insignificant at a 5% level from 2013 to 2016; that is, the average TGR in the upper-middle income cities was slightly higher than in the high-income cities from 2013 to 2016.

#### Average efficiency indicators in the high income and upper middle-income cities

Table 5 compares the average CO_2_, AQI, energy consumption, the average GDP, respiratory disease, mortality rate and health expenditure efficiencies in the high-income and upper-middle income cities.

**Table 5:** Average indicator efficiencies in the high-income and upper-middle income cities

City	2013					2014					
	CO_2_	AQI	Respiratory	Energy	Healthcare expenditure	CO_2_	AQI	Respiratory	Mortality	Energy	Healthcare expenditure
High-income	0.814	0.837	0.801	0.788	0.491	0.741	0.972	0.884	0.875	0.760	0.530
Upper-middle income	0.659	0.799	0.848	0.653	0.461	0.659	0.938	0.805	0.813	0.649	0.460

City	2015					2016					
	CO_2_	AQI	Respiratory	Energy	Healthcare expenditure	CO_2_	AQI	Respiratory	Mortality	Energy	Healthcare expenditure
High-income	0.793	0.716	0.866	0.793	0.356	0.665	0.882	0.840	0.869	0.665	0.451
Upper-middle income	0.668	0.636	0.855	0.652	0.373	0.522	0.803	0.839	0.841	0.522	0.454

Except for 2015, the average CO_2_ emissions and AQI were lower in the high-income cities than in the upper-middle income cities. The average CO_2_ emissions efficiency in the high-income cities declined from 0.81 in 2013 to 0.67 in 2016, and in the upper-middle income cities, first rose from 0.66 in 2013 to around 0.67 in 2015 and then fell to 0.52 in 2016; therefore, the average CO_2_ emissions efficiencies between the city groups narrowed slightly in 2016. In general, however, the high-income cities needed to strengthen their CO_2_ emissions management and consider coordinating their CO_2_ and air pollutant emissions management.

The AQI efficiency in the high-income cities increased from 0.84 in 2013 to 0.97 in 2014, fell to 0.71 in 2015, then rebounded to 0.89 in 2016, and in the upper-middle income cities increased from 0.80 in 2013 to 0.94 in 2014, declined in 2015 to 0.67, and rebounded slightly to 0.80 in 2016; therefore, the average AQI efficiency gap widened between the two city groups.

While the high-income cities had higher energy consumption efficiency than the upper-middle income cities, this fluctuated down from around 0.89 in 2013 to around 0.67 in 2016. The energy consumption efficiency in the upper-middle income cities also fluctuated down from 0.65 in 2013 to around 0.52 in 2016. Therefore, improvements were needed in both city groups. These results were similar to the empirical results in Li et al. [29] in which it was found that the high-income cities had higher efficiencies than the upper-middle income cities.

The average health expenditure efficiencies in both city groups were low, with the average in the high-income cities being 0.49 and the average in the upper- middle income cities being 0.461. In 2014, the average health expenditure efficiency in the high-income cities rose to around 0.53, but in the upper-middle income cities fell slightly to below 0.46. In 2015, the health expenditure efficiency in the high-income cities fell sharply to 0.36 and in the upper-middle income city average health expenditure efficiency fell to 0.37. In 2016, both city groups had significant increases to around 0.45, with the upper-middle income cities being slightly higher than the high-income cities.

The average mortality rate efficiency gap between the two city groups also narrowed. The average mortality rate efficiency rose in the high-income cities from 0.81 in 2013 to 0.84 in 2016, but dropped in the upper-middle income cities from around 0.88 in 2013 to 0.84 in 2016.

While the average healthcare expenditure efficiency in the high-income cities was slightly higher than in the upper-middle income cities, it was declining in both city groups, reaching its lowest in 2015. The average healthcare expenditure efficiencies in the high-income cities declined faster over the 4 years, and by 2015 and 2016, was lower than in the upper-middle income cities; therefore, significant improvements were needed across the board.

#### Average efficiency indicators by region

Table 6 shows the main annual average input and output indicator efficiencies in each region.

**Table 6:** Average efficiencies from 2013–2016 by region

City	CO_2_				AQI			
	2013	2014	2015	2016	2013	2014	2015	2016
East	0.784	0.761	0.816	0.796	0.867	0.976	0.81	0.917
Central	0.764	0.693	0.695	0.478	0.71	0.934	0.587	0.818
Northeast	0.821	0.787	0.793	0.457	0.577	0.948	0.387	0.783
West	0.642	0.621	0.645	0.499	0.885	0.946	0.671	0.798

City	Respiratory				Mortality			
	2013	2014	2015	2016	2013	2014	2015	201H
East	0.82	0.867	0.902	0.888	0.838	0.896	0.932	0.917
Central	0.856	0.799	0.957	0.853	0.872	0.745	0.929	0.87
Northeast	0.677	0.574	0.542	0.524	0.64	0.546	0.516	0.516
West	0.855	0.906	0.856	0.872	0.889	0.917	0.902	0.877

City	Energy				Healthcare expenditure			
	2013	2014	2015	2016	2013	2014	2015	2016
East	0.783	0.752	0.8	0.796	0.59	0.654	0.475	0.563
Central	0.698	0.696	0.687	0.453	0.356	0.292	0.247	0.374
Northeast	0.785	0.827	0.793	0.457	0.177	0.292	0.114	0.265
West	0.642	0.619	0.636	0.517	0.526	0.525	0.409	0.454

The highest regional average carbon dioxide emissions efficiency in 2013 and 2014, was in the northeast region and the lowest was in the western region, and in 2015 and 2016, the highest carbon dioxide emissions efficiency was in the eastern region, and the lowest efficiencies were in the western and northeastern regions. From 2013 to 2015, the annual average carbon dioxide emissions efficiency in the western region was the lowest of all four regions, but in 2016, was only lower than the eastern region. Regardless, the average carbon dioxide emissions efficiency in the western region was fluctuating and declining, and there was a great need for improvement.

The regional average AQI index efficiency in the eastern region was higher than in the other four regions, and in 2013, 2014 and 2015, the AQI index efficiency was higher than the central and northeastern regions. Except for 2014, the average AQI index efficiency in the northeastern region was the lowest of the four regions and in 2015 was only 0.387, and although there was a significant increase to 0.78 in 2016, there was a great need for improvement. In the western region, the average AQI index efficiency in 2013 was the highest of the four regions. It increased significantly in 2014 but was still slightly lower than the eastern region, and then began to decline significantly. Although it rebounded in 2016, it was lower than in the eastern and central regions. The average AQI index efficiency in the central region was low in 2013 and 2015, was the lowest of the four regions in 2014, but was still high at 0.934, but in 2016 was higher than in the western and northeastern regions. Overall, the average AQI index efficiencies in the western and northeastern regions had a slight fluctuating upward trend.

The average annual respiratory diseases efficiency was the lowest in the northeastern region, and was only 0.524 in 2016. The average respiratory diseases efficiencies in the eastern, central, and western regions, however, were relatively close.

The northeast region had the lowest average mortality rate efficiency, which except for 2014, was only around 0.52 in the other 3 years. The average mortality rate efficiency in the eastern region was the highest of the four regions in 2015 and 2016 at above 0.9, the western region had the highest average mortality rate efficiency in 2013 and 2014 at above 0.85, and the average mortality rate efficiency in the central region ranked second in 3 years, but was ranked third in 2014.

The energy consumption efficiency was high in all regions. The energy consumption efficiency in the northeast region was the highest in 2013 and 2014, was slightly lower than the eastern region in 2015 and was lower than both the eastern and western regions in 2016. The average energy consumption efficiency in the western region from 2013 to 2015 was the worst at below 0.65. In the eastern region, the energy consumption efficiency was slightly lower than in the northeast in 2013 and 2014 but was the highest in 2015 and 2016. The energy consumption efficiency in the central region continued to decline and by 2016 was only 0.453; therefore, there was significant need for improvements.

The health expenditure efficiencies were low in all regions. The health expenditure efficiency in the eastern region was higher than in other regions but in 2016, was only 0.56. The health expenditure efficiency in the northeast region was lower than 0.3 in all 4 years and only 0.27 in 2016, and in the central region was only slightly higher. The health expenditure efficiency in the western region was slightly higher than that in the northeast and central regions, but in 2016, was only about 0.45.

Of the six above-mentioned indicators, the average health expenditure efficiencies in each region were the lowest, followed by the energy consumption and carbon dioxide emissions efficiencies. Therefore, future regional governance attention needs to be paid to health expenditure, energy consumption and carbon dioxide efficiency improvements.

The AQI index, respiratory disease, mortality, and energy consumption efficiencies in the northeastern region were the lowest of the four regions for most of the years; therefore, governance in these areas needs attention.

While the eastern region had higher AQI index, mortality rate and health expenditure efficiencies than the other regions, the health expenditure efficiency needed significant improvements.

The average energy consumption efficiency in the western region was low and while the health expenditure ranked second to the eastern region, governance improvements are still needed.

## Discussion

Using a modified two stage Undesirable Meta Dynamic Network model, this study jointly analyzed energy consumption, economic growth, air pollution and health treatment expenditure data from 31 Chinese high-income and upper-middle income cities from 2013 to 2016, from which the following conclusions were made found:

(i) The high-income cities had higher average efficiencies than the upper-middle income cities. Of the 10 eastern region high-income cities, Guangzhou and Shanghai had average efficiencies of 1, with the least efficient being Shijiazhuang. In the other regions, the upper-middle income cities required greater technology and health treatment investments. These results indicated that the environmental and health expenditure efficiencies of the more economically developed regions were significantly higher than in the less developed regions. Economically developed areas are mainly located in the eastern coastal areas of China, most of which have relatively good weather conditions and the economic capacity to support healthy investment and development.

(ii) Guangzhou, Lhasa, Nanning, and Shanghai had production stage efficiencies of 1, and Guangzhou, Lhasa, Nanning, Shanghai and Fuzhou had healthcare resource utilization stage efficiencies of 1. The average production stage efficiencies in most cities were higher than the healthcare resource utilization stage efficiencies; therefore, greater efforts are needed to improve the healthcare resource utilization efficiencies. Guangzhou and Shanghai were the most economically developed cities in China in terms of economic strength and social development and achieved the best production and environmental efficiencies. Lhasa, which is is dominated by tourism, has a sparse population, and few high-polluting industries, had good environmental production stage and health efficiencies. Fuzhou, which is located in the coastal area of southeastern China, has good diffusion conditions and a high economic level, had the most efficient production, environmental and health input efficiencies.

(iii) The technology gap ratios (TGRs) in the high-income cities were slightly higher than in the upper-middle income cities, indicating that the high-income cities had slightly higher technological levels. Therefore, the upper-middle income cities need to learn from the high-income cities to improve their general TGR.

(iv) While the high-income cities had higher energy consumption efficiencies than the upper-middle income cities, this was decreasing in most cities. There were insignificant respiratory disease efficiency differences between the high-income and upper-middle income cities; however, the high-income cities had decreasing mortality rate efficiencies while the upper-middle income cities had increasing mortality rate efficiencies. Overall, most cities needed to strengthen their health governance to ensure balanced economic growth and urban expansion.

(v) The average AQI efficiencies in both the high-income and upper-middle income cities were higher than the average CO_2_ efficiencies. However, the high-income cities had lower average CO_2_ emissions and AQI index efficiencies than the upper-middle income cities, and the AQI efficiency differences between the two city groups was expanding. As most cities were focusing on air pollution controls rather than carbon dioxide emissions controls, greater efforts are needed to coordinate the air pollution and carbon dioxide emissions treatments.

In summary, climate change, economic growth and social development all pose great challenges to development. Therefore, to effectively respond to these challenges and problems, governments need to actively adapt measures to local conditions, develop scientific governance systems, and formulate short, medium- and long-term dynamic strategic management directions. Therefore, the following policy suggestions are given.

(1) Hospital and medical system reform is needed and a wide-ranging medical management system developed that has local medical systems based on regional characteristics and local disease characteristics to ensure a complete governance system from prevention to emergency response to supervision to security. For example, for areas that need to deal with the air pollutant discharges such as Zhengzhou, the focus should be on strengthening investment in the prevention and treatment of the diseases caused by air pollution and especially by PM_2.5_. Investment should also be strengthened in labor and the materials to ensure active prevention, active response and effective supervision and control.

(2) Local governments need to strengthen awareness in adolescents and residents about the health problems and diseases caused by air pollution. There is a large local education gap in China, with the high-income cities generally having better education than the upper-middle income cities; therefore, the education and policy promotions in the upper middle-income cities needs to be improved. Due to regional differences, different cities need to take different measures.

(3) High-income cities need to take action based on their specific problems and challenges. Overall, healthcare governance requires medium and long-term response measures and coordinated governance responses to reduce the incidence of respiratory diseases and the associated mortality.

(4) Of the 17 upper-middle income cities, Hangzhou, Changchun, Harbin, Chengdu, Guiyang, Kunming and Xi’an need to prioritize healthcare governance efficiency by establishing sound medical management systems and emergency environmental pollution treatments. For example, while Hangzhou is a coastal city that is conducive to the discharge of harmful air pollutants, industrial environmental pollution has adversely affected resident health. Therefore, the city needs to put more emphasis on healthcare treatment and air pollution treatment investments. Chengdu is in a basin surrounded by mountains, which is not conducive to the discharge of air pollutants; therefore, medical monitoring should be prioritized, and in addition to strengthening medical monitoring governance in Kunming, the treatment of carbon dioxide emissions should take precedence over the treatment of air pollutants.

(5) As high-income cities have more advanced technologies and fewer polluting industries, the CO_2_ and AQI emissions treatments have achieved some good results. However, upper-middle income cities such as Lanzhou, Taiyuan and Shijiazhuang, all of which have high energy consumption and high polluting industry structures, need to focus on reducing their CO_2_ and AQI emissions. Therefore, the local governments need to adapt the treatment controls to local conditions and design medium to long-term development strategies.

(6) Upper-middle income cities need to actively learn from the technology governance experiences in the more efficient higher-income cities. Based on their city’s economic development stage, economic resources and industry structures, local and regional governments need to develop health governance models that meet their own characteristics, and when reforming their current medical systems, high-income cities could learn from countries that have good medical systems such as Japan and the United Kingdom. For example, special attention should be given to developing and employing competent medical workers. Education programs for adolescents and residents about the health problems linked to air pollution could help them take environmental protection action

## Conclusion

This study used DEA to determine the input and output improvements needed in 31 Chinese cities and to propose corresponding policy recommendations. Climate change, economic growth and social development all pose great challenges to human development. Therefore, to effectively respond to these challenges and problems, the government needs to actively adapt measures to local conditions, develop scientific governance systems, and formulate short, medium- and long-term dynamic strategic management directions. All regions should take active measures based on local meteorological conditions, geological characteristics, resource endowments and population characteristics to improve their energy consumption, economic, environmental and health efficiencies.

As the sample data were obtained from publicly released Chinese government data, the data used in this research were the latest available. Therefore, future research could continue to track the air pollution, economic growth and health governance efficiencies based on updated data and provide the central and local governments with more timely and effective policy recommendations and management decision-making references.

## Data Availability

Not applicable.

## Appendices

### Appendix 1

See Table 7.

**Table 7:** Overall efficiency by city and region from 2013–2016

DMU	Cities by income	Region	2013	2014	2015	2016	Average
Beijing	High-income city	East	1.000	0.951	0.528	0.502	0.745
Fuzhou	High-income city	East	0.892	0.823	0.803	0.809	0.832
Guangzhou	High-income city	East	1.000	1.000	1.000	1.000	1.000
Hangzhou	High-income city	East	0.460	0.569	0.465	0.472	0.492
Haikou	Upper-middle income city	East	0.813	0.767	0.758	0.757	0.774
Jinan	High-income city	East	0.368	0.414	0.373	1.000	0.539
Nanjing	High-income city	East	0.463	0.620	0.504	0.510	0.524
Shanghai	High-income city	East	1.000	1.000	1.000	1.000	1.000
Shijiazhuang	Upper-middle income city	East	0.258	0.279	0.230	0.212	0.245
Tianjin	High-income city	East	0.399	0.474	0.406	0.392	0.418
Changchun	Upper-middle income city	Northeast	0.470	0.498	0.444	0.358	0.442
Harbin	Upper-middle income city	Northeast	0.429	0.462	0.422	0.329	0.411
Shenyang	High-income city	Northeast	0.386	0.480	0.361	0.457	0.421
Changsha	High-income city	Central	0.479	0.675	0.560	0.649	0.591
Hefei	Upper-middle income city	Central	0.434	0.468	0.492	0.880	0.568
Nanchang	High-income city	Central	0.510	0.516	0.460	0.351	0.459
Taiyuan	Upper-middle income city	Central	0.354	0.193	0.295	0.317	0.290
Wuhan	High-income city	Central	0.917	0.541	0.465	0.399	0.581
Zhengzhou	High-income city	Central	0.515	0.531	0.517	0.416	0.495
Chengdu	Upper-middle income city	West	0.369	0.414	0.375	0.339	0.374
Chongqing	Upper-middle income city	West	0.278	0.336	0.279	0.518	0.352
Guiyang	Upper-middle income city	West	0.323	0.341	0.315	0.292	0.318
Huhehot	High-income city	West	0.457	0.586	0.470	0.441	0.489
Kunming	Upper-middle income city	West	0.390	0.386	0.332	0.293	0.350
Lanzhou	Upper-middle income city	West	0.421	0.391	0.270	0.379	0.365
Lhasa	Upper-middle income city	West	1.000	1.000	1.000	1.000	1.000
Nanning	Upper-middle income city	West	1.000	1.000	1.000	0.681	0.920
Urumqi	Upper-middle income city	West	0.912	0.923	0.680	0.823	0.835
Xian	Upper-middle income city	West	0.393	0.413	0.360	0.314	0.370
Xining	Upper-middle income city	West	0.347	0.386	0.320	0.289	0.336
Yinchuan	Upper-middle income city	West	0.690	0.574	0.464	0.568	0.574

### Appendix 2

See Table 8.

**Table 8:** Production efficiency and healthcare resource utilization efficiencies from 2013–2016

DMU	Cities by income	Region	2013(I)	2013(II)	2014(I)	2014(II)	2015(I)	2015(II)	2016(I)	2016(II)
Beijing	High-income city	East	1.000	1.000	1.000	0.901	1.000	0.055	0.948	0.056
Fuzhou	High-income city	East	0.784	1.000	0.647	1.000	0.606	1.000	0.618	1.000
Guangzhou	High-income city	East	1.000	1.000	1.000	1.000	1.000	1.000	1.000	1.000
Haikou	High-income city	East	0.625	1.000	0.533	1.000	0.517	1.000	0.514	1.000
Hangzhou	Upper-middle income city	East	0.775	0.145	0.805	0.333	0.818	0.113	0.772	0.172
Jinan	High-income city	East	0.553	0.183	0.645	0.182	0.606	0.139	1.000	1.000
Nanjing	High-income city	East	0.743	0.183	0.752	0.487	0.885	0.123	0.849	0.171
Shanghai	High-income city	East	1.000	1.000	1.000	1.000	1.000	1.000	1.000	1.000
Shijiazhuang	Upper-middle income city	East	0.361	0.155	0.367	0.191	0.346	0.115	0.318	0.106
Tianjin	High-income city	East	0.741	0.057	0.738	0.210	0.731	0.082	0.722	0.062
Changchun	Upper-middle income city	Northeast	0.770	0.169	0.797	0.198	0.811	0.077	0.606	0.109
Harbin	Upper-middle income city	Northeast	0.743	0.114	0.769	0.156	0.775	0.068	0.539	0.119
Shenyang	High-income city	Northeast	0.625	0.148	0.625	0.335	0.611	0.110	0.545	0.368
Changsha	High-income city	Central	0.761	0.197	0.781	0.568	0.779	0.341	0.739	0.559
Hefei	Upper-middle income city	Central	0.709	0.159	0.743	0.194	0.730	0.253	0.759	1.000
Nanchang	High-income city	Central	0.824	0.196	0.827	0.204	0.798	0.121	0.549	0.152
Taiyuan	Upper-middle income city	Central	0.337	0.371	0.334	0.052	0.332	0.257	0.350	0.284
Wuhan	High-income city	Central	0.833	1.000	0.811	0.271	0.771	0.160	0.688	0.110
Zhengzhou	High-income city	Central	0.878	0.151	0.890	0.172	0.917	0.117	0.695	0.138
Chengdu	Upper-middle income city	West	0.626	0.112	0.615	0.213	0.653	0.097	0.581	0.097
Chongqing	Upper-middle income city	West	0.498	0.057	0.504	0.167	0.509	0.048	0.503	0.533
Guiyang	Upper-middle income city	West	0.402	0.243	0.479	0.204	0.497	0.132	0.413	0.170
Huhehot	High-income city	West	0.599	0.316	0.760	0.413	0.748	0.193	0.620	0.263
Kunming	Upper-middle income city	West	0.465	0.316	0.459	0.313	0.517	0.146	0.434	0.153
Lanzhou	Upper-middle income city	West	0.359	0.484	0.366	0.416	0.325	0.214	0.375	0.383
Lhasa	Upper-middle income city	West	1.000	1.000	1.000	1.000	1.000	1.000	1.000	1.000
Nanning	Upper-middle income city	West	1.000	1.000	1.000	1.000	1.000	1.000	1.000	0.361
Urumqi	Upper-middle income city	West	0.823	1.000	0.847	1.000	0.573	0.787	0.646	1.000
Xian	Upper-middle income city	West	0.636	0.149	0.653	0.174	0.625	0.095	0.486	0.141
Xining	Upper-middle income city	West	0.299	0.395	0.311	0.462	0.290	0.350	0.324	0.255
Yinchuan	Upper-middle income city	West	0.473	0.906	0.450	0.698	0.399	0.528	0.440	0.696
